# Referral to the national network of integrated care: the nurses’ perception

**DOI:** 10.1590/1518-8345.3800.3372

**Published:** 2020-10-19

**Authors:** Susana Alexandra Fonseca-Teixeira, Pedro Parreira, Lisete Mónico, Anabela Salgueiro-Oliveira, João Costa Amado

**Affiliations:** 1 Universidade Católica Portuguesa, Instituto de Ciências da Saúde, Porto, Portugal.; 2 Escola Superior de Enfermagem de Coimbra, Unidade de Pesquisa em Ciências da Saúde: Enfermagem, Coimbra, Portugal.; 3 Universidade de Coimbra, Faculdade de Psicologia e Ciências da Educação, Coimbra, Portugal.; 4 Universidade Católica Portuguesa, Centro de Investigação Interdisciplinar em Saúde, Instituto de Ciências da Saúde, Porto, Portugal.

**Keywords:** Home Care Services, Quality of Health Care, Long-Term Care, Patient Care Team, Nursing, Portugal, Serviços de Assistência Domiciliar, Qualidade da Assistência à Saúde, Assistência de Longa Duração, Equipe de Assistência ao Paciente, Enfermagem, Portugal, Servicios de Atención de Salud a Domicilio, Calidad de la Atención de Salud, Cuidados a Largo Plazo, Grupo de Atención al Paciente, Enfermería, Portugal

## Abstract

**Objective:**

to understand the referral to the National Network of Integrated Continuous Care, from the perspective of nurses who work in this care context.

**Method:**

an exploratory and descriptive study with a qualitative approach, with data collection between July and September 2019 through interviews with 12 nurses who work in Integrated Continuous Care Teams, in Northern Portugal. The content analysis technique was used to analyze the statements.

**Results:**

the professionals revealed that there are difficulties and constraints in the process of referring users to the National Network of Integrated Continuous Care. The process is bureaucratic, complex, and time-consuming, conditioning user accessibility to timely care.

**Conclusion:**

the referral process is a very bureaucratic and time-consuming procedure, which not only conditions and delays users’ access to the National Network of Integrated Continuous Care network, contributing to the worsening of the clinical status of some patients. The number of professionals is insufficient, inducing the demand for services through urgency. The focus on primary care should seek to improve inequalities in access, compete for more equitable and accessible care, generating more quality in health care.

## Abstract (pt)

**Objetivo:**

compreender a referenciação para a Rede Nacional de Cuidados Continuados Integrados, a partir da perspetiva de enfermeiros que atuam nesse contexto assistencial.

**Método:**

estudo exploratório, descritivo com abordagem qualitativa, cujos dados foram coletados entre julho e setembro de 2019, por meio de entrevistas a 12 enfermeiros que trabalham em Equipes de Cuidados Continuados Integrados, do norte de Portugal. Para analisar as falas, utilizou-se a técnica de análise de conteúdo.

**Resultados:**

os profissionais revelaram que existem dificuldades e constrangimentos no processo de referenciação dos usuários para a Rede Nacional de Cuidados Continuados Integrados. O processo é burocrático, complexo e demorado, condicionando a acessibilidade dos usuários aos cuidados em tempo útil.

**Conclusão:**

o processo de referenciação é um procedimento muito burocratizado e demorado, que condiciona e atrasa o acesso dos usuários à Rede Nacional de Cuidados Continuados Integrados, contribuindo para o agravamento do estado clínico de alguns pacientes. O número de profissionais é insuficiente, induzindo à procura de serviços pela via da urgência. A aposta na atenção primária deverá procurar melhorar as desigualdades no acesso, concorrer para cuidados mais equitativos e acessíveis gerando mais qualidade nos cuidados de saúde.

## Abstract (es)

**Objetivo:**

comprender la derivación a la Red Nacional de Cuidados Continuados Integrados, desde la perspectiva de los enfermeros que actúan en este contexto asistencial.

**Método:**

estudio exploratorio, descriptivo, con enfoque cualitativo, cuyos datos fueron recolectados entre julio y septiembre de 2019, a través de entrevistas con 12 enfermeros que trabajan en Equipos de Cuidados Continuados Integrados, del norte de Portugal. Para analizar las declaraciones, se utilizó la técnica de análisis de contenido.

**Resultados:**

los profesionales revelaron que existen dificultades y limitaciones en el proceso de derivación de usuarios a la Red Nacional de Cuidados Continuados Integrados. El proceso es burocrático, complejo y demorado, condicionando la accesibilidad del usuario a la atención en tiempo oportuno.

**Conclusión:**

el proceso de derivación es un procedimiento muy burocrático y demorado, que no solo condiciona y atrasa el acceso de los usuarios a la Red Nacional de Cuidados Continuados Integrados sino que además contribuye al agravamiento del estado clínico de algunos pacientes. El número de profesionales es insuficiente, lo que induce a la demanda de los servicios de la emergencia. La apuesta en la atención primaria debe orientarse a mejorar las desigualdades en el acceso, competir por una atención más equitativa y accesible y, así, brindar una mayor calidad en la atención de la salud.

## Introduction

Demographic evolution is characterized by a sharp increase in the older adult population, which is associated with a higher prevalence of chronic conditions and dependence, thus increasing the demand for long-term care^(^ 1 ^-^ 2 ^)^. This situation has also led to an increase in the health costs^(^ 2 ^-^ 3 ^)^.

The National Network of Integrated Continuous Care (“*Rede Nacional de Cuidados Continuados Integrados”*, RNCCI, in Portuguese) in Portugal, beginning in 2006, aimed at creating integrated responses of social action and health with regard to continuous care following good European practices in this area. However, despite being considered an effective strategy, it shows an insufficient response capacity, representing less than 30% of the current needs and, even less of, the expected future needs^(^ 4 ^-^ 6 ^)^. In this context, the Integrated Continuous Care Teams (*“Equipes de Cuidados Continuados Integrados”*, ECCI, in Portuguese) are of particular importance since, in addition to contributing to the increase in responses to a national need, they present themselves as a solution for closer care. Increasing the capacity of these teams is assumed to be an incomparably lesser financial investment effort compared to the increase in the number of inpatient units^(^ 4 ^,^ 7 ^-^ 8 ^)^. For this reason, in recent years, there has been a strong international interest in the development of resolute and cost-effective interventions to support older adults living at their homes, reducing the demand for hospital and institutionalized care^(^ 9 ^-^ 11 ^)^.

A number of studies on the quality of health care also point out that an understanding of the organizational issues in the provision of health services is essential to explain variations in care and progress towards continuous quality improvement. Health systems need to maximize their effectiveness and efficiency and those of long-term care, provide continuous assistance in service delivery, and adapt to the needs of the patients. With the growing economic and financial demand, health professionals are challenged to show that the care provided is of high quality, adequate, appropriate, efficient and effective, producing the best results in the patients^(^ 6 ^,^ 12 ^-^ 13 ^)^. Hence, the shift to a health system focused on individual needs and preferences allows for a new insight into the impact of these policies^(^ 10 ^,^ 14 ^)^. The primary care network must become more robust, respond to people’s needs and coordinate with other services^(^ 10 ^,^ 15 ^)^.

Based on this framework, this article is conceptually based on the Nursing Role Effectiveness Model (NREM)^(^ 16 ^)^ which is anchored in Donabedian’s Health Quality Model, developed from three concepts: the structure, the process, and the results with regard to quality assistance^(^ 16 ^)^. The NREM allows for assessing the contribution of nurses to health care, advocating a set of relationships between the variables of structure, process, and results. The structural variables refer to issues related to organizational resources, such as the availability of equipment and the skills of the health care team; the process integrates nurses’ interventions and the results those changes in the patient’s health status as a result of care provision^(^ 17 ^)^. The characteristics in the structure and in the nurses’ intervention process will provide changes in the results. The structure integrates factors related to the patient, the nurse, and the organization. The process integrates autonomous and interdependent interventions, such as team communication, case management, and care coordination. The results consider functional state, self-care, symptom control, and adverse effects^(^ 16 ^)^.

This research aims to understand the referral to the National Network of Integrated Continuous Care, from the perspective of nurses who work in this care context.

## Method

The research carried out was exploratory and descriptive with a qualitative approach, since it was intended to know the studied phenomenon, *i.e.*, the experiences of the participants, based on the descriptions from the surveyed individuals^(^ 18 ^)^.

A convenience sample was constituted with twelve nurses from the ECCI in the northern region of Portugal, having as inclusion criterion performing functions in the ECCI for more than 3 years. For the definition of the number of participants, the data saturation criterion was adopted, with the end of the collection phase as the empirical framework of the research was outlined to achieve the objective. One of the researchers went to the units to carry out the interviews that took place between July and September 2019, during the work period, on a day and time scheduled according to the availability of the participants and with institutional consent.

The 12 nurses targeted by the research were aged between 36 and 60 years old (M=46 years old and SD=7.5). The time of professional experience varied between 13 and 36 years (M=24 years and SD=7.5) and the experience in ECCI varied between 5 and 10 years (M=8 years and SD=1.7).

The data collection method used was the semi-structured interview^(^ 19 ^-^ 20 ^)^, through a thematic script whose themes addressed the difficulties and constraints in the patient referral process for the RNCCI network, so a specific order was not followed in conducting the interview, allowing for the conversation to continue and for the spontaneity of the participant^(^ 19 ^-^ 20 ^)^, while maintaining a focus on the predefined objective. All the interviews were recorded on audio, respecting the ethical assumptions underlying research of this nature. The interviews lasted a mean of 40 minutes and were later transcribed in full to a *Word* document on the researcher’s private computer and analyzed using the content analysis technique^(^ 21 ^-^ 22 ^)^aiming at refining subjective content descriptions, in order to objectively highlight the nature and relative strengths of the stimuli. Such a path allows for the emergence of valid and replicable inferences from the data to the context under study^(^ 23 ^)^. In this way, the transcribed interviews were read several times to familiarize with the data and to facilitate understanding of the whole.

Content analysis was performed using NVivo^®^, respecting the three phases proposed by Bardin^(^ 21 ^-^ 22 ^)^: pre-analysis, data exploration, and treatment of the results, including inference and interpretation processes. Thus, these stages were followed: full reading of open answers, definition of analysis units taking into account the registration units and the enumeration units, and designation of categories, subcategories, and answer indicators. As references, the units of recording and enumeration were considered^(^ 24 ^)^. The meaning units were extracted, condensed, and labeled with a code according to the objective of the study and organized according to the NREM conceptual model (structure, process, and results). This approach aimed to ensure reliability and to maintain a balance between the authors’ prior understanding of the referral process and to assess how the relationship between structure and process variables impacts on the results^(^ 25 ^)^.

This study was initiated after approval by the Ethics Committee of the Health Center Groups in the northern region of Portugal. All the interviews were conducted in a reserved place, and the signing of the Free and Informed Consent Term was requested, according to the Declaration of Helsinki^(^ 26 ^)^, and the anonymity of the participants was ensured by assigning an identification code with the letter E (“*Enfermeiro*” in Portuguese) and a number from 1 to 12.

## Results

From analyzing the content of the transcribed interviews, three categories emerged. The “human and material resources” category was the one with the highest number of references (registration and enumeration units) with 26.78% of the total references, followed by the “difficulties and constraints in the referral process” (13.04%) and the “information system” (11.41%) categories.

### Human and Materials Resources

In the “human and material resources” category, the testimonials revealed that, regarding assistance responses, the number of professionals in the ECCI is insufficient (namely physicians and nurses). The majority refer that the teams have an insufficient number of nurses. The workload of the physicians is variable in the ECCI, for the same number of vacancies allocated, the number of hours of medical care is different, and the majority reported being insufficient, as explained in the excerpts of the interviews: *Sometimes people have to be referred to the emergency department because there is no medical response* (E1); *(…) there is no medical support (…) cannot respond due to lack of resources (…)* (E1); *(…) we couldn’t respond (…) because the team is very small (…)* (E8); *(…) given their profile, nursing care hours are also scarce (…)*(E*5*); *(…) there are other times when the team becomes perfectly insufficient given the time consumption and the severity of the patients B* (E3).

The participants also considered that, for a more effective response, it would be important to include other professionals, namely physical therapists, nursing attendants, speech therapists, and occupational therapists, as mentioned in the excerpts of the interviews: *The professionals are not enough (…) a nurse with more time available (…) other professionals from other fields like physiotherapists would be important* (E11)*; (…) we would have room for an occupational therapist (…) speech therapist (…)* (E3); *(…) the nursing attendant has always been an asset (…)* (E10).

Associated with the lack of human resources, there are also other constraints, such as the lack of transportation for commuting, as mentioned in the excerpts of the interviews: *The vehicles [car] are one of the biggest constraints we have (…)* (E12); *(…) car (…) is insufficient (…)* (E5); *(…) the biggest constraint will be the car [vehicle] (…)* (E9).

It was also mentioned that the opening of the units and the number of allocated vacancies are not adjusted to the needs of the covered population, as shown in the excerpts of the interviews: *They should (…) adjust the vacancies because (…) our advice because it is smaller (…) wouldn’t need so many (…)* (E11); *(…) more beds were needed (…) if we had more resources (…)* (E5).

### Information System

In the Information Systems (“*Sistemas de Informação*”, SI, in Portuguese) category, the testimonies revealed that the registration modules of the SI-RNCCI application are complex and extremely time-consuming. They mentioned that there is irrelevant information and inadequate or not applicable scales for some patients that hinder the adequate elaboration of the processes. They pointed out that the information described in the notes is often incomplete and inconsistent and that some records are controversial. The participants considered that the requirement to record on two overlapping platforms - SI-RNCCI and SClínico - implies the need to duplicate records and makes referencing less rapid and less easy. They also stressed that it is sometimes difficult to obtain data referring to the family, namely when data on financial income are requested, as shown in the excerpts of the interviews: *(…) It’s extremely bureaucratic, extremely time-consuming and very difficult (…) to collect family data because (…) they don’t want to give data and have to sign the consent form (…) record in the two systems [SI-RNCCI and SClínico] (…) it’s a waste of time (…)not recording what is essential (…)* (E1); *A lot of time is spent making records and referrals are worse (…) the information of SClínico doesn’t migrate to SI-RNCCI* *(…) some scales are not correct (…) not applicable (…) for certain patients (…) we have difficulty analyzing the notes, some information is missing, (…)very sparse, we have to go to the (…) Electronic Health Record and other systems (…)* (E2); *if there was no need to duplicate to the SI-RNCCI (…) the information that (…) is found (…) in the SClínico (…) the referencing could be faster and (…) easier* (E3); *In the application we are unable to visualize the patient’s needs and do not always correspond to the reality (…)* (E4); *It is a very bureaucratic system (…)* (E7); *(…) it should be (…) more simplified* (E8); *The fact that we have to record (…) the same thing in the two systems [SI-RNCCI and SClínico] (…) is complex (…) it takes a long time, some scales make no sense and (…)there are one or two parameters that, strictly speaking, cannot even be completed for some patients (…). It’s not easy to view the previous records* (E12).

### Referral Process

The participants stated that, in detriment of the referral for the patients to the ECCI, they were referred to the inpatient services of the RNCCI network, due to the fact that the patients need continuous care and the ECCI schedules are not adequate to their real needs, such as elucidated in the excerpts of the interviews: *Sometimes people have to be referred to the emergency department because there is no medical response* (E1); *(…) in terms of scheduling, they are not suited to what the real needs are, the real needs of patients are not covered with work from 8 am to 8 pm* (E1); *(…) palliative cares (…) from 8 pm to 8 am the next day (...) it’s a long time without support (…)* (E12).

The referral of the patients also depends on the assessment of the multidisciplinary team, consisting of a physician, a nurse, and a senior social service technician. The team needs to proceed with the preparation of a medical, nursing, and social service report to be presented to the Local Coordinating Team and subsequently validated by the Regional Coordinating Team. Only after completing this procedure will the patient be able to enter an RNCCI unit.

The results of the present research showed that referring patients to RNCCI is a very bureaucratic, complex and time-consuming process. Teams are not motivated to refer and, sometimes, do not start or delay the process and, in others, after starting the referral process, they give up or choose only to refer extremely serious cases. They also mention that those in the health units are the most unmotivated and reluctant professionals and that some even refuse to refer because it is a long process. They describe that the Functional Units *(“Unidades Funcionais”,* UF, in Portuguese) can take two months or more to carry out the referral process and that, in the hospitals, the time it takes to complete the report and validate by the competent authorities often leads to a postponement of the patient’s transfer for a week. As it is a time-consuming process, hospitals discharge the patients to their homes and ask the physician of the health units to make referrals, which is a more difficult and time-consuming process. Sometimes, when the process is restarted again, patients no longer meet the necessary criteria, missing out on opportunities in referral, as shown in the excerpts of the interviews: *(…) they say that it’s very bureaucratic (…) there’s no medical availability to record (…)* (E1); *This procedure is highly bureaucratic and is one of the main factors (…) conditioning the patient’s accessibility to the RNCCI* (E3); *The time it takes to complete the report (…) often motivates the patient’s discharge to be postponed (…)a week and therefore the hospital gives up the referral* (E3)*; “(…) process is so complicated to reference that (…) they end up demotivating (…)* (E2); *They don’t refer, they just don’t do it, other times they only refer cases that are extremely severe and that don’t even fit in the ECCI environment (…)* (E1); *Often as the doctors are not always accessible (…)”* (E4); *(…) it’s enough for the doctor to say no (…) despite the patient needs (…) and the patient doesn’t come in”* (E12); *(…) due to the bureaucracy (…) they will delay and postpone the referral (…)* (E10); *(…)often (…) having the process started at the RNCCI, but (…) some documentation is needed (…) they end by giving up the referral and (…) sending the patients to their homes (…)* (E1); *(…) due to the waiting time, they send us to (…) the homes (…)* (E6)*; Making a note to the doctor of the health unit alerting to the need for the patient having to be referred to the RNCCI (…)*(E3); *Delaying to the point that there’s no longer referencing in time for what is intended for the patient, namely (…) the palliative cares (…)* (E3).

According to the participants, the teams that refer the most are family health units, to the detriment of the hospitals and the RNCCI inpatient units. There is a lack of knowledge about the ECCI and the type of response provided. Most of the referred patients are social cases, claiming rehabilitation criteria in the vast majority of cases. They also point out that many of the referred patients have a serious clinical condition, with several comorbidities, unstable and, therefore, do not fall within the scope of the ECCI, as mentioned in the excerpts of the interviews: *The vast majority of the patients arrive via a UF (…)* (E1); *The hospital doesn’t refer (…) out of ignorance (…)*(E10); *(…) the patient (…) comes with an indication for physical therapy (…) we don’t have physiotherapy care (…)* (E1); *(…)the majority has more to do with the lack of resolution of social problems than with the treatment of ulcers or the management of the therapeutic regimen.* (E1); *(…) the rehabilitation criteria are used (…) for referencing (…)* (E1); *(…) when the patient at the ECCI (…) the reason for the need for care does not always match* (E1); *(…) when the patient arrives at our unit, care no longer has anything to do with the initial need, it’s usually much worse* (E5); *They are usually patients with a very weak state (…) with many comorbidities* (E1); *Most of the patients* *(…) are not clinically stabilized* (E1); *(…) come to the ECCI due to lack of response from other units (…)* (E1); *(…) they only refer cases that are extremely severe and that don’t even fall within the scope of the ECCI (…)* (E1)*.*

Finally, other aspects mentioned that make it difficult to refer and/or transfer patients to inpatient units are based on the limited number of vacancies, on geographical dispersion and on economic criteria, as explained in the excerpts of the interviews: *(…) the economic criterion is a barrier for families, the geographical dispersion of the units, namely here in the northern region is also another barrier* (E5); *(…) families always prefer units close to their area of residence, which do not always exist or are not always available with the adequate type for the patient* (E1); *(…)the patients are at their homes waiting for a long time because of the location they choose* (E8).

Other constraints mentioned, which make it impossible for the patient to enter the ECCI, were the following: the absence of the ECCI and health unit physician’s inter-substitution on vacation periods, not having an informal caregiver, and the documents being out of date, as mentioned in the excerpts of the interviews: *(…) the ECCI doctor during the holiday period has inter-substitution (…), but (…) the doctor who is replacing (…) is also on holidays during that period (…)* (E2); *(…) it is often not possible to refer the patient just for the simple fact (…) for not having a citizen card (…) the health unit doctor is on holidays (…)* (E12); *(…) when the doctors are on holidays there is no one to replace them (…)* (E1).

Data show that there are clearly constraints in the referral and articulation process for the RNCCI network, which conditions the accessibility of the patients to long-term care, contributing to the worsening of their clinical status.

## Discussion

The categories that emerged from the analysis of the content of the interviews and the mobilization of the NREM model allow for disclosing aspects related to problems in the structure (S), difficulties alluding to the referencing process (P) with influence on the results (R), also showing a difficulty in articulation in the relations between (S-P-R). The analysis of the relationship among the variables related to structure (human and material resources) and process (information system) showed to have an impact on the results, generating difficulties and constraints in the referral process culminating in loss in the effectiveness and efficiency of users at the RNCCI network. The difficulties displayed in the referencing structure (S) and process (P) culminate in a notable limitation due to lack of access to nursing care (R). It will be urgent to intervene in such factors to create more organized and efficient processes and less bureaucratic, as they are generators of users’ inaccessibility to nursing cares. Some research studies carried out within the scope of long-term care based on the NREM indicates that the nurses’ experience (structure) and the independent nursing interventions (process) were significantly related to functional status, symptom control and better patient clinical outcomes (results)^(^ 16 ^,^ 27 ^)^.

The results of the present research show that referencing is a very bureaucratic and time-consuming (process) procedure, which substantially conditions and delays the accessibility of the patients to the RNCCI network, preventing the patients from having access to timely care (results). This constraint is aggravated by the fact that it is mandatory to integrate three different professionals, requiring validation by two entities (one local and another regional), further aggravated by the mandatory validation by social security, regarding the determination of exemptions and payment of fees. The bureaucratic process between the UF and the local coordination teams prevents the process from being more fluid and swift, conditioning the patient’s referral time for too long, thus jeopardizing the fulfillment of the objectives proposed for the user. Furthermore, the fact that the registration modules of the SI-RNCCI application are complex and time-consuming and the obligation to record on two platforms (SI-RNCCI and SClínico) makes it difficult to prepare the processes. The delay in the referral process leads to occupancy rates in the suboptimized ECCI, generating serious inefficiencies in the users’ access to long-term care. The fact that this procedure is highly bureaucratic and of great complexity conditions the accessibility of the patients to the RNCCI network. The respondents pointed out as one of the possible causes for hospitals to under-refer to the RNCCI network and to refer patients to their homes the fact that they do not prolong hospital stay and, consequently, reduce the costs of hospital care. They state that this procedure causes a delay in the patient’s admission in due time due to the constraints of the UF. The constraints referred to in this study were also referred to by other studies that assess patient accessibility to the RNCCI network^(^ 28 ^-^ 31 ^)^.

The delay in the referral process, in addition to conditioning the patients’ admission, also worsens their clinical condition so, at the time of admission, the necessary response may no longer fall within the scope of the ECCI. In addition, associated with the insufficient number of professionals, namely physicians, they culminate in the lack of close monitoring of the patients, which leads to the demand for emergency services. The results of this study are corroborated by other authors who report that patients without a close follow-up resort to the emergency department more frequently, despite the fact that hospitalizations are not only expensive, but are also potentially harmful to the health of the older population^(^ 31 ^-^ 34 ^)^, therefore contrasting with the recommendation of the Organization for Economic Cooperation and Development, which estimates that 30% of the hospital activity in Portugal could be done in the community^(^ 3 ^)^.

The results of this research demonstrated regional inequalities in access to health care provided by the ECCI, which is why it is necessary to adjust the number of vacancies allocated according to population density, as well as to increase human and logistical resources, namely transportation, so that there is greater equity between access to health care and more efficient use. These results are also corroborated by some studies that report that there is an inequity in access to the RNCCI network, and regional geographic dispersion with some degree of randomness due to its location. In addition to being more accessible due to proximity, ECCI also generate lower financial costs, although they continue to be largely underutilized throughout the country^(^ 3 ^-^ 4 ^,^ 35 ^-^ 36 ^)^. Thus, we consider it important to increase the awareness of the hospitals and of the primary care network for the referral of patients to the ECCI.

Now we verify that health care currently takes place in a fragmented environment, which is why it is necessary to provide continuous and coordinated care centered on people^(^ 36 ^-^ 37 ^)^ where accessibility to the ECCI is an advantageous option. Promoting equity in access to health care and a more efficient use of the available resources are fundamental aspects for improving the health status of the population, especially the older adults. Increasing the response capacity of the ECCI is assumed to be an incomparably lesser financial investment effort due to the increase in the number and types of hospitalization, so that more attention should be placed on primary care, as it improves the quality of health care and reduces inequalities in access, generating more equitable and accessible cares. Such bet is reflected, in addition to the health results in the population, in a reduction in the total cost of care, obtaining results at a more accessible and sustainable cost^(^ 3 ^,^ 5 ^,^ 15 ^)^. Health systems need to maximize their effectiveness and efficiency of health services and long-term care, providing continuous care between services, providing important improvements in the quality and accessibility of the users, adapting to their care needs^(^ 5 ^,^ 13 ^-^ 15 ^)^.

A number of studies carried out in the context of outpatient care have shown that nursing interventions have contributed to improving the results of the patients^(^ 27 ^)^. NREM has the potential to identify specific results generated by the care provided by nurses, highlighting the role and responsibility of the Nursing profession, and justifying its importance in making decisions about health policies^(^ 16 ^)^.

The research allowed identifying and understanding the difficulties and constraints in the referral process of patients who need long-term care to the RNCCI network. It also contributes to assist the management in decision making, namely in the improvement of strategies and tools with a contribution to ensuring the improvement of quality in the continuity of care and the reduction in the number complications and in the worsening of the user’s health due to lack of care because of non-referrals.

Although the research has achieved its objective, it is understood as a possible limitation the fact that the research took place only in the northern region, which does not mean that the reality presented is similar in other regions of Portugal, being unwise to generalize the results.

## Conclusion

The results of this research revealed that the referral process is a very bureaucratic and time-consuming procedure, which substantially conditions and delays the accessibility of the patients to the RNCCI network, preventing the users from having access to care in a timely manner. The lack of professionals is evident, which creates difficulties in monitoring the patients’ proximity, thus inducing the demand for emergency services. The delay in the referral process, in addition to being able to condition the patients’ admission, also worsens their clinical conditions. The health team professionals highlighted the need for systematic and timely access to records.

Promoting equity between access to health care and its more efficient use, taking into account the available resources, is a fundamental aspect for improving the health status of the population, especially the older adults. Thus, it is considered crucial to create conditions to improve the structural (increase the response capacity of the ECCI, namely with the reinforcement of professionals) and process (simplify the referral process, enable systematic and timely access to data, and improve interoperability in information systems improving care monitoring and evaluation) variables to obtain better results (fewer inequalities of access, more equitable and accessible care). Thus, this research presents itself as a humble but valid contribution to the improvement in the referral and quality of health care to be provided to the users.
